# Improving the hydrothermal stability of zeolite Y by La^3+^ cation exchange as a catalyst for the aqueous-phase hydrogenation of levulinic acid †

**DOI:** 10.1039/d0ra08907a

**Published:** 2021-01-29

**Authors:** Hue-Tong Vu, Michael Goepel, Roger Gläser

**Affiliations:** Institute of Chemical Technology, Universität Leipzig Linnéstr. 3 04103 Leipzig Germany roger.glaeser@uni-leipzig.de

## Abstract

La^3+^ cation exchange is shown to improve the hydrothermal stability and catalytic activity of bifunctional zeolite Pt/Y catalysts in the aqueous-phase hydrogenation of levulinic acid (LA) with formic acid (FA) as hydrogen source. La^3+^ cation exchange of zeolite Y (*n*_Si_/*n*_Al_ = 16) was conducted both in aqueous solution and in the solid state. The hydrothermal stability of La^3+^-containing zeolite Y probed by exposure to the reaction mixture (0.2 mol L^−1^ LA, 0.6 mol L^−1^ FA) at 473 K under autogenous pressure for 24 h improves with increasing La content. The material exhibiting the highest La content (0.5 mmol g^−1^) is the most stable with a preservation of 25% of the initial specific micropore volume after the hydrothermal treatment, whereas unmodified zeolite Y completely loses its microporosity. A new procedure using DRIFTS is a useful supplementary tool for quantifying the framework degradation of Y-type zeolites after hydrothermal treatment. Bifunctional Pt/Y catalysts after La^3+^ cation exchange are more active than the parent Y-zeolite for the hydrogenation of LA to γ-valerolactone (GVL), with significant enhancements in LA conversion, *i.e.*, 94% *vs.* 42%, and GVL yield, *i.e.*, 72% *vs.* 34%., after 24 h.

## Abstract 2

La^3+^ cation exchange is shown to improve the hydrothermal stability and catalytic activity of bifunctional zeolite Pt/Y catalysts in the aqueous-phase hydrogenation of levulinic acid (LA) with formic acid (FA) as hydrogen source.

## Graphical abstract

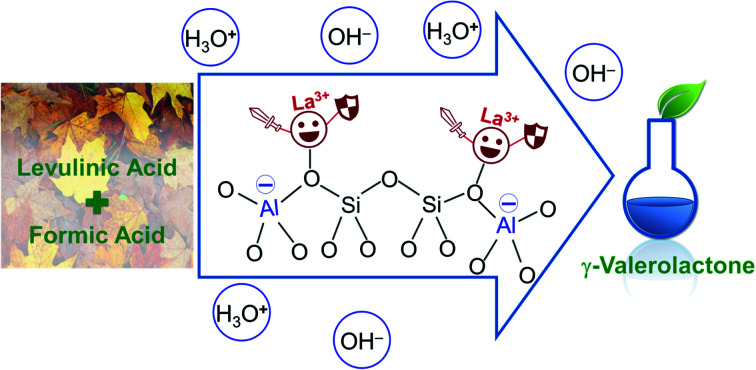

## 1. Introduction

Lanthanum cation exchange, conducted both in liquid phase (mostly water) and in solid state, has been extensively studied and applied for improving the hydrothermal stability of zeolite Y (Y) in fluid catalytic cracking (FCC), typically operated in the gas phase at moderate to high temperatures, *e.g.*, 573–1073 K.^1–4^ An important advantage of La^3+^ cation exchange is the high preservation of both the crystalline structure and textural properties of zeolite Y after ion exchange either in the aqueous phase^5–11^ or in the solid state.^7^ The incorporation of La^3+^ cations into zeolite Y has been proven to increase its stability in FCC. This retards undesired dealumination during catalyst regeneration, which is facilitated under the typical regeneration conditions, *i.e.*, at 973 K in the presence of steam generated through coke combustion.^12,13^ In addition, La^3+^ cation exchange of faujasitic zeolites was also reported to enhance the catalytic activity for acid-catalyzed reactions, *e.g.*, isomerization, hydroisomerization and cracking, owing to the presence of “super acid sites” arising from the interaction between Si–OH–Al and La^3+^ cations in their vicinity.^14–16^ Most studies, however, deal with gas-phase applications of La^3+^ cation-exchanged zeolite Y, while its hydrothermal stability in liquid water at elevated temperature (*T* ≥ 473 K) and under catalytic conditions is still not well understood.

**Scheme 1:**
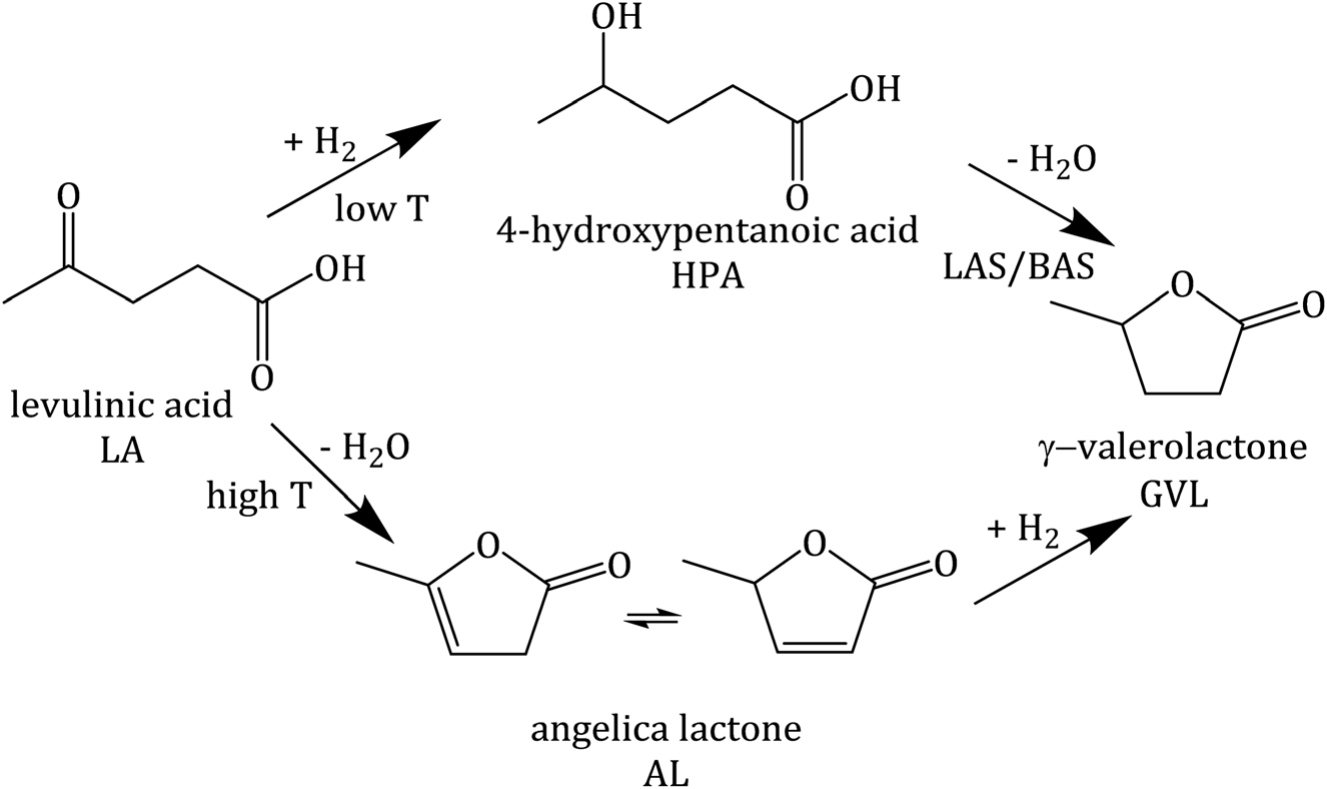
Reaction pathway of hydrogenation of levulinic acid (LA), adapted from ref. 40.

Zeolites are considered to be promising catalysts for aqueous-phase processing (APP) of biomass,^17–21^ in which water is used as a “green solvent”, typically at elevated temperature *T* ≥ 473 K and pressure *p* ∼4 MPa.^22–27^ One prominent example among numerous conversion in APP is the aqueous-phase hydrogenation of biomass-derived levulinic acid (LA) to γ-valerolactone (GVL) using formic acid (FA) as an internal hydrogen source.^28–33^ LA and FA are both products formed with an equimolar ratio from cellulose hydrolysis.^34,35^ GVL is a versatile platform chemical which can serve as a “green solvent”,^36^ a fuel additive^37^ or an intermediate to the valeric biofuel family^38^ and a series of fine chemicals.^39^ The conversion of LA to GVL entails a metal-catalyzed hydrogenation and an acid-catalyzed dehydration (Scheme 1).^40^ Thus, bifunctional catalysts containing a de-/hydrogenation functionality, *e.g.*, Pt, Ru or Pd, and an acid functionality, *e.g.*, zeolites, activated carbon or alumina, are required.

A previous investigation using Pt supported on zeolite Y (Pt/Y) revealed that under the applied reaction conditions (0.2 mol L^−1^ LA, 0.6 mol L^−1^ FA, *T* ≥ 473 K, *p* ∼4 MPa, 24 h) zeolite Y undergoes a complete loss of crystallinity. This contributed to the comparatively low catalytic activity (LA conversion (*X*_LA_) of 42%).^41^ In the same study, surface silylation of zeolite Y was applied to largely prevent desilication and zeolite framework disruption under APP-related conditions. A significant drawback of this approach is the deterioration of the textural properties (loss of specific surface area by 48% and specific pore volume by 33%) by silylation compared with parent zeolite Y. This results in a drastic decrease in catalytic activity in the hydrogenation of LA (GVL yield (*Y*_GVL_) of 12% compared with 34% for unmodified Pt/Y).

To overcome these disadvantages, La^3+^ cation exchange is studied here for stabilization of the zeolite framework *via* charge compensation and, thus, strengthening Al–O bonds, while preserving crystallinity and textural properties of zeolite Y. Therefore, the current work aims at investigating the influence of La^3+^ cation exchange (in aqueous solution or in the solid state) on the hydrothermal stability and the catalytic activity of bifunctional Pt/zeolite Y catalysts in the aqueous-phase hydrogenation of LA in the presence of FA. The effects of La^3+^ cation exchange and silylation are compared in terms of the stabilizing effect on structure of zeolite Y under APP-related conditions. Additionally, a new procedure using diffuse reflectance infrared Fourier transformation spectroscopy (DRIFTS) was examined as a supplementary tool for quantifying the deterioration degree of the zeolite Y framework after exposure to APP-related conditions.

## 2. Experimental section

### 2.1 Materials

Zeolite Y (Y) (CBV 720, H^+^ form, *n*_Si_/*n*_Al_ = 16) was supplied by Zeolyst International. Levulinic acid (LA, ≥ 98%) was purchased from by Merck Schuchardt OHG. Lanthanum(iii) nitrate hexahydrate (La(NO_3_)_3_·6H_2_O, 99.99%), formic acid (FA, 99–100%) and γ-valerolactone (GVL, 99%) were obtained from Sigma-Aldrich. Pentanoic acid (PA, 99%), sodium nitrate (NaNO_3_, 99.0%) and tetraammine platinum(ii) nitrate (Pt(NH_3_)_4_(NO_3_)_2_, 99.99%) were purchased from Alfa Aesar. All chemicals were used as received without further purification.

### 2.2 Catalyst preparation

#### 2.2.1 La^3+^ cation exchange in the liquid phase

The commercial zeolite Y (H^+^ form) was stirred in an aqueous solution of 0.5 mol L^−1^ NaNO_3_ with a liquid-to-solid ratio of 10 cm^3^ g^−1^ at 323 K for 24 h. Subsequently, the solid was separated by centrifugation, washed three times with deionized water (10 cm^3^ water per g of zeolite) and dried at 373 K for 24 h in ambient air. The dried material was transferred to a crucible and thermally treated with a heating ramp of 5 K min^−1^ under N_2_ (10 cm^3^ min^−1^) at 393 K for 6 h and consecutively at 773 K for 4 h resulting in the Na^+^-form of zeolite Y (Na–Y). In order to achieve a high La^3+^ cation exchange degree, Na–Y was used as starting material for the La^3+^ cation exchange in the liquid phase as Na–Y is more susceptible to La^3+^ cation exchange.^42,43^ In a typical La^3+^ cation exchange step, a suspension of zeolite Na–Y in an aqueous 0.5 mol L^−1^ La(NO_3_)_3_ solution (liquid-to-solid ratio = 10 cm^3^ g^−1^) was stirred at 373 K for 4 h. Afterwards, the solid was separated, washed, dried and calcined following the same procedure as for the Na^+^ cation exchange described above. The La^3+^ cation exchange step including drying and calcination was repeated twice to obtain a higher La^3+^ cation exchange degree. The obtained materials were labelled as *x*La,Na–Y(lq) with *x* referring to the molar ratio *n*_La_/*n*_Al_ determined by EDX analysis and “lq” signifying that the La^3+^ cation exchange was carried out in the liquid phase. With the assumption that each La^3+^ cation compensates three negative framework charges (AlO_4_^−^), the La^3+^ cation exchange degree was calculated as1
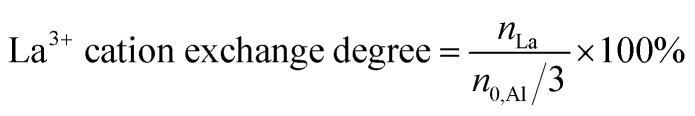
with *n*_0,Al_ being the Al content of the parent zeolite Y determined by ICP-OES (*n*_0,Al_ = 1 mmol g^−1^). *n*_La_ is the La content (in mmol g^−1^) of the La^3+^ cation-exchanged zeolite Na–Y (determined by EDX analysis) assuming that all introduced La^3+^ cations compensate three negative charges of the zeolite framework.

#### 2.2.2 Solid-state La^3+^ cation exchange

A physical mixture of 3 g zeolite Y and La(NO_3_)_3_·6H_2_O was ground in a mortar for 30 min. The initial molar ratio of La and Al was varied (0.15, 0.3, 0.6, 1.2) and the obtained materials were named *x*La-Y(ss) with *x* being the molar ratio *n*_La_/*n*_Al_ determined by EDX analysis and “ss” referring to the solid-state La^3+^ cation exchange. The ground mixture was thermally treated using the same procedure as for the Na^+^ cation exchange described above in Chapter 2.2.1. Afterwards, the obtained materials were washed three times with 30 cm^3^ of deionized water, separated by centrifugation and subsequently dried at 373 K for 24 h in ambient air. The La^3+^ cation exchange degree was calculated as given in eqn (1) (*cf.* Chapter 2.2.1).

#### 2.2.3 Loading with Pt by incipient wetness impregnation

In a typical preparation for an aimed Pt content of 3 wt%, a solution of 0.061 g Pt(NH_3_)_4_(NO_3_)_2_ in 0.75 cm^3^ deionized water was added dropwise to 1 g of (La^3+^ cation-exchanged) zeolite Y. The resulting solid was then dried at 373 K for 12 h and subsequently calcined at 723 K with a heating rate of 5 K min^−1^ in static air for 4 h.

### 2.3 Catalyst characterization

Diffuse reflectance infrared Fourier transform spectroscopy (DRIFTS) was carried out in a Bruker Vector 22 FTIR solid phase spectrometer equipped with a heated DRIFTS cell with a ZnSe window. Prior to the measurement, the samples were dried at 573 K for 30 min under an N_2_ flow of 100 cm^3^ min^−1^. Using the same atmosphere, spectra were recorded at 373 K in the range of 500–4000 cm^−1^, by addition of 100 scans and with a nominal resolution of 4 cm^−1^. For pyridine adsorption experiments, the cell was cooled and kept at 363 K, which was followed by saturation of the zeolite with gaseous pyridine in an N_2_ flow (100 cm^3^ min^−1^) for 1 h. In order to remove physisorbed pyridine, the sample was flushed with N_2_ (100 cm^3^ min^−1^) at 363 K for 2 h. For recording the spectrum, the temperature was increased to 473 K and held for 30 min. The bands located at 1445 cm^−1^ and 1554 cm^−1^ are respectively ascribed to Brønsted (BAS) and Lewis acid sites (LAS).^44^ The intensity of these two bands (1445 cm^−1^ and 1554 cm^−1^) were used to calculate the ratio of BAS-to-LAS.

The acid site density (ASD) and acid site strength distribution of zeolite Y before and after modification were determined by temperature-programmed desorption of ammonia (TPD-NH_3_) using a tubular glass microreactor coupled with a mass spectrometer (MS, Pfeiffer GSD 301). 50 mg of zeolite powder were placed in the glass reactor and heated with a ramp of 5 K min^−1^ under helium flow (30 cm^3^ min^−1^) to 573 K, at which the temperature was held for 1 h for drying. After cooling down to 363 K, the sample was flushed with NH_3_*via* 6 successive pulses of 1 cm^3^ until saturation. Subsequently, physisorbed NH_3_ was removed by treatment at 363 K under the same He flow. This was followed by heating up to 823 K with a ramp of 10 K min^−1^. The amount of desorbed NH_3_ was quantified using the MS fragment *m*/*z* = 15. Depending on the temperature at maximum NH_3_ desorption (*T*_des_), acid sites are classified into 3 groups, *i.e.*, strong (*T*_des_ ≥ 750 K), moderate (500 K < *T*_des_ < 750 K) and weak (*T*_des_ ≤ 500 K) acid sites as proposed for Y-type zeolites.^45^

A micromeritics ASAP2010 sorption analyzer was used to record N_2_ sorption isotherms at 77 K. The samples were evacuated at 523 K under vacuum pressure of 3 × 10^−11^ MPa for 6 h prior to the measurements. The specific surface area *A*_BET_ was determined by the Brunauer–Emmett–Teller (BET) model. The specific pore volume *V*_P_ was estimated from the N_2_ uptake at a relative pressure *p p*_0_^−1^ of 0.99. The specific micropore volume *V*_micro_ was calculated using the *t*-plot model. The specific mesopore volume (*V*_meso_) determined as *V*_meso_ = *V*_P_ − *V*_micro_.

The element content was determined by optical emission spectrometry with inductively coupled plasma (ICP-OES) using a PerkinElmer Optima 8000. Prior to the analysis, the samples were dissolved in 2.0 cm^3^ HF, 3.0 cm^3^ HNO_3_ and 3.0 cm^3^ HCl and diluted to obtain aqueous solutions which also contained 12.0 cm^3^ H_3_BO_4_ for complexation of excessive HF.

Powder X-ray diffraction (XRD) patterns were recorded at room temperature using a Siemens D5000 diffractometer. The diffraction intensity of Cu-Kα radiation (*λ* = 0.154 nm) was measured in the range of 2*θ* between 4° and 90°, with a step size of 0.005° and a counting time of 0.2 seconds. The full width at half maximum of the reflex at 2*θ* = 15.9° is referred to as FWHM_15.9_.

Energy dispersive X-ray (EDX) spectroscopy was performed on a LEO 1530 Gemini (Zeiss, Germany, acceleration voltage 20 kV) equipped with an EDX detector from Oxford Instruments (Model 7426). EDX spectra were evaluated with the INCA software, developed by ETAS. The mean element content, *e.g.*, *n*_La_, *n*_Al_, *n*_Na_, *n*_Si_, and the corresponding standard deviation, *e.g.*, Δ*n*_La_, Δ*n*_Al_, Δ*n*_Na_, Δ*n*_Si_, were calculated using results for 3 different particles (3 point scans for each particle). Afterwards, the *n*_La_/*n*_Al_-ratio was determined using the mean element content, and the error of the molar ratio, *e.g.*, Δ(*n*_Si_/*n*_Al_), was calculated following the rule of uncertainty propagation as2
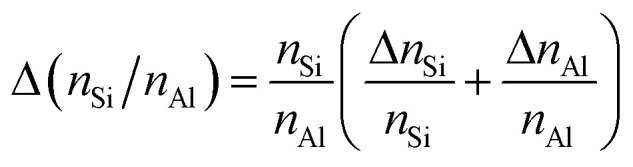


Transmission electron microscopy (TEM) was conducted using a JEOL JEM-2100 Plus instrument. Zeolites were ground, suspended in ethanol, and segregated for 10 min. A few drops of this suspension were placed on a lacey carbon copper grid. Ethanol was evaporated at room temperature. High angle annular dark field scanning transmission electron microscopy (HAADF-STEM) imaging was carried out at 200 kV using a beam current of 12 μA.

### 2.4 Investigation of the hydrothermal stability

Stability tests were conducted for the zeolites Y, Na–Y and the La^3+^ cation-exchanged zeolites. The reactant solution for the stability tests and for the catalytic experiments (*cf.* Section 2.5) was identical, *i.e.*, a 5 wt% aqueous solution of LA (0.2 mol L^−1^) and FA (0.6 mol L^−1^). In a stainless-steel autoclave with a 60 cm^3^ polytetrafluoroethylene (PTFE) liner, 1 g of the zeolite, dried at 373 K under static air for 12 h prior to the experiment, was added to 40 cm^3^ of the reactant solution. The sealed autoclave was kept in an oven at 473 K for 24 h. After rapid cooling the autoclave to room temperature, the solid was separated by centrifugation, and subsequently washed three times with 30 cm^3^ of deionized water. The obtained solid was dried at 373 K in static air for 24 h and subsequently analyzed by XRD, N_2_ sorption and DRIFTS.

### 2.5 Catalytic hydrogenation of levulinic acid with formic acid

Catalytic experiments were carried out in a 300 cm^3^ stainless steel batch reactor (Model #4560, *Parr Instruments Company*) with an overhead stirrer, a heater and an external monitor (Model # 4848, *Parr Instruments Company*) for controlling temperature, pressure and stirring speed. Prior to the catalytic experiments, each catalyst was pressed, crushed and sieved to obtain particles with an average grain size < 200 μm. Subsequently, the catalysts were externally reduced using a tubular oven at 673 K under a flow of H_2_ (2.0 cm^3^ min^−1^) in N_2_ (8.0 cm^3^ min^−1^) for 4 h. In a typical catalytic experiment, 0.50 g reduced catalyst and 125 cm^3^ of the reaction solution (*cf.* Chapter 2.4) were loaded into the reactor. The reactor was sealed, purged with N_2_ three times and heated to 493 K. The reaction was performed at 493 K under autogenous pressure for 24 h while stirring at 700 min^−1^.

Liquid samples were withdrawn at the start of the reaction, *i.e.*, when the desired temperature was reached, typically after 15 min, and after 6, 12 and 24 h of reaction. The withdrawn samples were filtered and characterized by high-performance liquid chromatography (HPLC). An HPLC system (Prominence-HPLC, Shimadzu, Kyoto, Japan) equipped with a photo-diode array detector and a Macherey-Nagel Nucleodur PolarTec column (4.6 × 250 mm) was used for the quantification of FA, LA, PA and GVL. An aqueous solution of 5 mmol L^−1^ H_2_SO_4_ was used as the mobile phase at a flow rate of 0.8 cm^3^ min^−1^ and the column was operated at 313 K. Quantification was conducted based on the intensity at a wavelength of 210 nm for FA, GVL and PA, and 226 nm for LA. Retention times of FA, LA, PA and GVL were determined using commercial samples of FA, LA, PA and GVL.

The conversion of FA (*X*_FA_), LA (*X*_LA_) and the yield of PA (*Y*_PA_), GVL (*Y*_GVL_) were calculated from the concentration of the compounds determined *via* external calibration of the respective integrated peak area.3
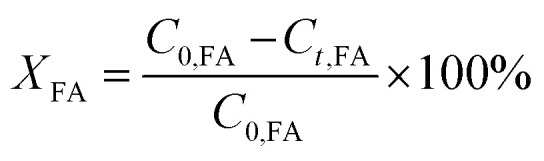
4
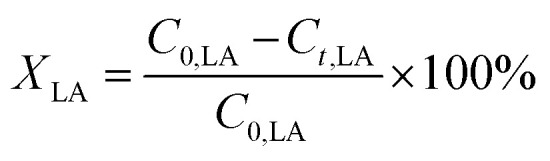
5
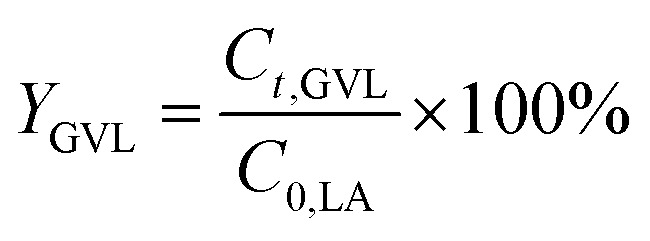
6
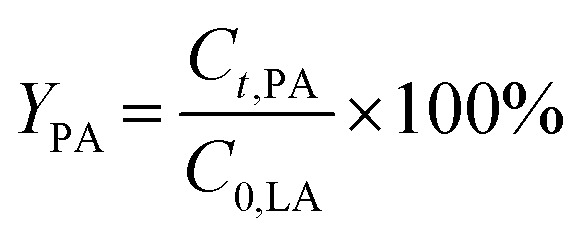
*C*_o,FA,LA_ refers to the initial concentration of FA, LA. *C*_t,FA,LA,PA,GVL_ denotes the concentration of FA, LA, PA and GVL after a specific reaction duration.

After the reaction, the reactor was cooled to room temperature, and the catalyst was removed by centrifugation, washed three times with 30 cm^3^ deionized water, dried in air at 373 K for 12 h and subsequently analyzed by XRD and N_2_ sorption. The carbon balance can only be closed to 80% due to the formation of humins strongly attached to the reactor wall.

## 3. Results and discussion

### 3.1 Ion exchange in the liquid phase *vs.* solid-state ion exchange

#### 3.1.1 Degree of La^3+^ cation exchange

Independent of the ion exchange procedure, La^3+^ cations were successfully incorporated into zeolite Y, which was demonstrated by the bulk La content determined by EDX analysis (Table 1, Fig. S1 and S2†).

**Table 1:** Specific La-content *n*_La_, *n*_Si_/*n*_Al_-ratio of the zeolites Y, Na–Y before and after La^3+^ cation exchange in the liquid or the solid state, determined by EDX analysis and La^3+^ cation exchange degree

Material	*n*_La_/mmol g^−1^	*n*_Si_/*n*_Al_	La^3+^ cation exchange degree/%
Y	n. d.^b^	16 ± 1^a^	n. d.
Na–Y	n. d.	15 ± 1^a^	n. d.
0.3La,Na–Y(lq)	0.19 ± 0.05	19 ± 2	62
0.6La,Na–Y(lq)	0.46 ± 0.04	20 ± 2	130
0.7La,Na–Y(lq)	0.32 ± 0.06	24 ± 2	104
0.1La–Y(ss)	0.08 ± 0.03	18 ± 2	21
0.2La–Y(ss)	0.16 ± 0.05	17 ± 2	45
0.3La–Y(ss)	0.23 ± 0.05	18 ± 2	65
0.7La–Y(ss)	0.50 ± 0.29	17 ± 2	140

a Determined by ICP-OES.

b n. d.: not determined.

In particular, after the first La^3+^ cation exchange step in the liquid phase, 0.3La,Na–Y(lq) was obtained with a La^3+^ cation exchange degree of 62%. The La^3+^ cation exchange degree reached 130% (0.6La,Na–Y(lq)) and dropped to 104% for 0.7La,Na–Y(lq) after two further successive ion exchange steps. This can be associated with the fact that Na–Y was saturated with La^3+^ cations after the second exchange step (*n*_La_ = 0.46 mmol g^−1^). Thus, the third ion exchange step did not improve the La^3+^ cation exchange degree, but rather caused leaching of La^3+^ cations resulting in a lower La content (*n*_La_ = 0.32 mmol g^−1^). Compared with the La^3+^ cation exchange degree for zeolite Y in the H^+^ form shown in Table S1,† the presence of Na^+^ cations in the zeolite Na–Y facilitates the La^3+^ cation exchange process as indicated by the much higher La^3+^ cation exchange degrees. For example, the La^3+^ cation exchange degree after the first ion exchange step using Na–Y is 62% for 0.3La,Na–Y(lq) with respect to 45% for 0.2La–Y(lq) without the Na^+^ cation exchange. The promoting effect of Na^+^ cations is well known and related to their weaker electrostatic attraction to the negative charges of the zeolite framework in comparison to H^+^.^7^

Demetallation (desilication and dealumination), a typical disadvantage of La^3+^ cation exchange in the liquid phase, is also observed here as evident from the increased *n*_Si_/*n*_Al_-ratios, *e.g.*, *n*_Si_/*n*_Al_ = 19, 20, and 24, after three sequential La^3+^ cation exchange steps starting from zeolite Na–Y (*n*_Si_/*n*_Al_ = 15). To quantify the extent of dealumination and desilication after La^3+^ cation exchange in the liquid phase, the concentration of Al^3+^ and Si^4+^ of the filtrate after each ion exchange step were determined by ICP-OES (*cf.* Table S2†) and the mass loss of Al and Si due to leaching after each La^3+^ cation exchange step was calculated (*cf.* Fig. S3†). Thus, loss of Al due to leaching after each of the three sequential La^3+^ cation exchange steps is 3.5, 0.6 and 0.1 wt%. These values are inversely linear with the *n*_Si_/*n*_Al_-ratio of the starting zeolite materials, *i.e.*, 15, 19 and 20, respectively. In contrast, the desilication increases from 0.2 over 0.3 to 0.4 wt% after the three sequential La^3+^ cation exchange steps. The leached Si weight percentage is not linearly correlated to the *n*_Si_/*n*_Al_-ratio of the starting materials. The desilication is more pronounced after the third La^3+^ cation exchange step. Despite the slight increase in *n*_Si_/*n*_Al_-ratio from 19 to 20, the weight percentage of leached Si increases significantly from 0.3 to 0.4 wt%. This is most probably a consequence of the dealumination which generates three new silanol groups Si–OH per Al atom removed. The hydrolysis of siloxane bonds (Si–O–Si) takes place at the newly generated Si–OH sites and, thus, facilitates further desilication.^46^ This finding is consistent with an earlier study on the La^3+^ cation exchange in the liquid phase of ultra-stabilized zeolite Y (H-USY, *n*_Si_/*n*_Al_ = 3.1) using a 0.5 mol L^−1^ La(NO_3_)_3_ aqueous solution.^7^

In contrast, no demetallation was observed after solid-state La^3+^ cation exchange. Also, the *n*_Si_/*n*_Al_-ratio remains unaffected after the ion exchange. With increasing the initial *n*_La_/*n*_Al_-ratio to 1.2, the highest La content is obtained for 0.7La–Y(ss), *i.e.*, *n*_La_ = 0.50 mmol g^−1^, which is comparable to 0.46 mmol g^−1^ (0.6La,Na–Y(lq)) the highest La content obtained from the La^3+^ cation exchange in the liquid phase. The higher standard deviation of the La content (Δ*n*_La_) determined by SEM-EDX for 0.7La–Y(ss), *i.e.*, Δ*n*_La_ = 0.29 mmol g^−1^, with respect to Δ*n*_La_ = 0.04 mmol g^−1^ (0.6La,Na–Y(lq)) or Δ*n*_La_ = 0.06 mmol g^−1^ (0.7La,Na–Y(lq)) is an indication of a lower homogeneity of local La distribution obtained after solid-state La^3+^ cation exchange. It was also observed that with increasing initial *n*_La_/*n*_Al_-ratio, the ion exchange efficiency, *i.e.*, the fraction of La^3+^ cations initially present in the ion exchange mixture incorporated into zeolite Y, decreased. As expected, the ion exchange efficiency is 53% for low initial *n*_La_/*n*_Al_ (≤0.3), *i.e.*, 0.1La–Y(ss) and 0.2La–Y(ss), and decreases to approximately 40% for both 0.3La–Y(ss) and 0.7La–Y(ss). This is due to the decreasing availability of exchange positions, *i.e.*, charge compensators of the negative charges of the zeolite framework AlO_4_^−^ determined by the Al content of the parent zeolite Y.

Interestingly, irrespective of the ion exchange procedure, a La^3+^ cation exchange degree > 100% was observed for highly ion-exchanged zeolites (*n*_La_ ≥ 0.32 mmol g^−1^), *i.e.*, 0.6La,Na–Y(lq), 0.7La,Na–Y(lq) and 0.7La–Y(ss). This observation is likely related to the complex nature of introduced La species. The incorporated La^3+^ cations are likely present not only as La^3+^ cations, but also as hydroxylated La^3+^ cations^7^ and as complexes^47^ formed through the dissociation of water in the coordination sphere of the La^3+^ cations as

7 La^3+^ + *n*H_2_O → (La(OH)_*n*_)^(3−*n*)+^ + *n*H^+^

or

8 2La^3+^ + *n*H_2_O → (La–O–La)^2(3−*n*)+^ + 2*n*H^+^

**Fig. 1:**
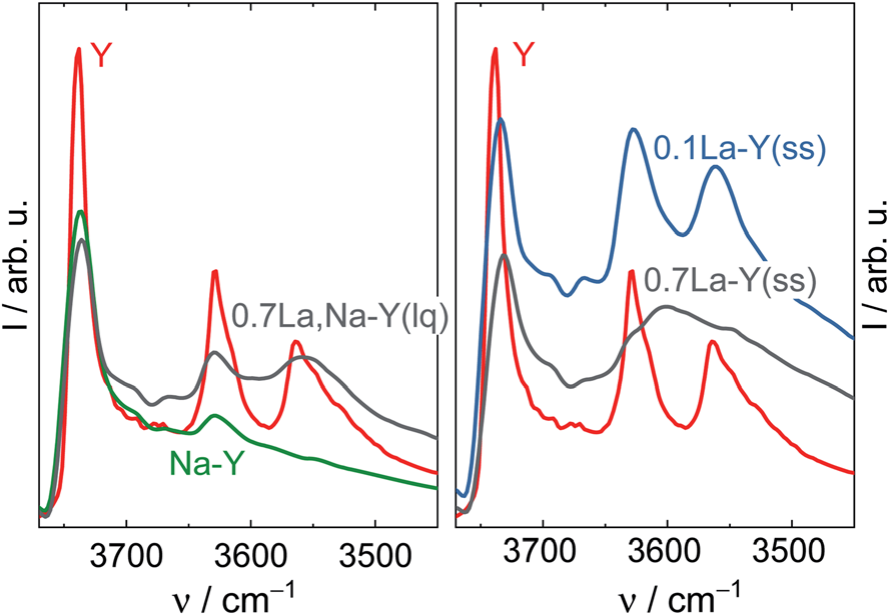
DRIFT spectra of the zeolites Y and Na–Y before and after La^3+^ cation exchange in the liquid phase (left) and in the solid state (right) recorded at 373 K.

According to the charge-compensating rule, one La^3+^ cation compensates the charges of three adjacent AlO_4_^−^ units. The existence of hydroxylated or complex species decreases the apparent lanthanum charge. Thus, La^3+^ cations are not necessarily restricted to the anticipated charge-compensation rule, but can also interact with two or only one AlO_4_^−^. Consequently, zeolite Y can accommodate an excess amount of La species. This is probably the case for the highly siliceous zeolite Y, in which the probability to find three AlO_4_^−^ sites in close proximity is scarce. The dissociation of water forming the needed OH^−^ species for the formation of hydroxylated La^3+^ cations and complexes is most likely favored in the presence of a high density of negative charged framework oxygen atoms. Furthermore, the presence of the various hydroxylated cations or complexes is evident from DRIFTS results (Fig. 1). The band corresponding to the bridging OH groups (Si–OH–Al) initially centered at 3562 cm^−1^ for the zeolites Y and Na–Y is observed at a lower wavenumber, *i.e.*, *ν* = 3558 cm^−1^, for 0.7La,Na–Y(lq) and with a broader shape. The red shift is probably because of the interaction between Si–OH–Al groups and the OH groups attached to extra framework La^3+^ cations which were previously reported at the wavenumber of 3556 cm^−1^.^48^ The broad shape is an indication of a complex mixture of interacting OH groups attached to La^3+^ cations.^13^ Since the La^3+^ cation exchange degree is calculated based on the bulk La content determined by EDX analysis without taking into account the nature of incorporated La, the La^3+^ cation exchange degree > 100% is observed for the zeolites with high La content (*n*_La_ ≥ 0.32 mmol g^−1^).

Nevertheless, to obtain highly ion-exchanged zeolites, the La^3+^ cation exchange in the solid state appears to be better suited in comparison to the ion exchange in the liquid phase as the involvement of a large quantity of expensive lanthanum salt (high La^3+^ cation exchange efficiency) and sequential ion exchange experiments are obsolete. More importantly, the undesired demetallation generating additional framework defects (silanol groups) is prevented in the solid-state ion exchange. On the other hand, at high La content (*n*_La_ > 0.50 mmol g^−1^), the comparatively low homogeneity of local La distribution indicated by a higher standard deviation of La content (Δ*n*_La_) determined by SEM-EDX is a potential disadvantage of the solid-state La^3+^ cation exchange.

#### 3.1.2 Structural properties

**Fig. 2:**
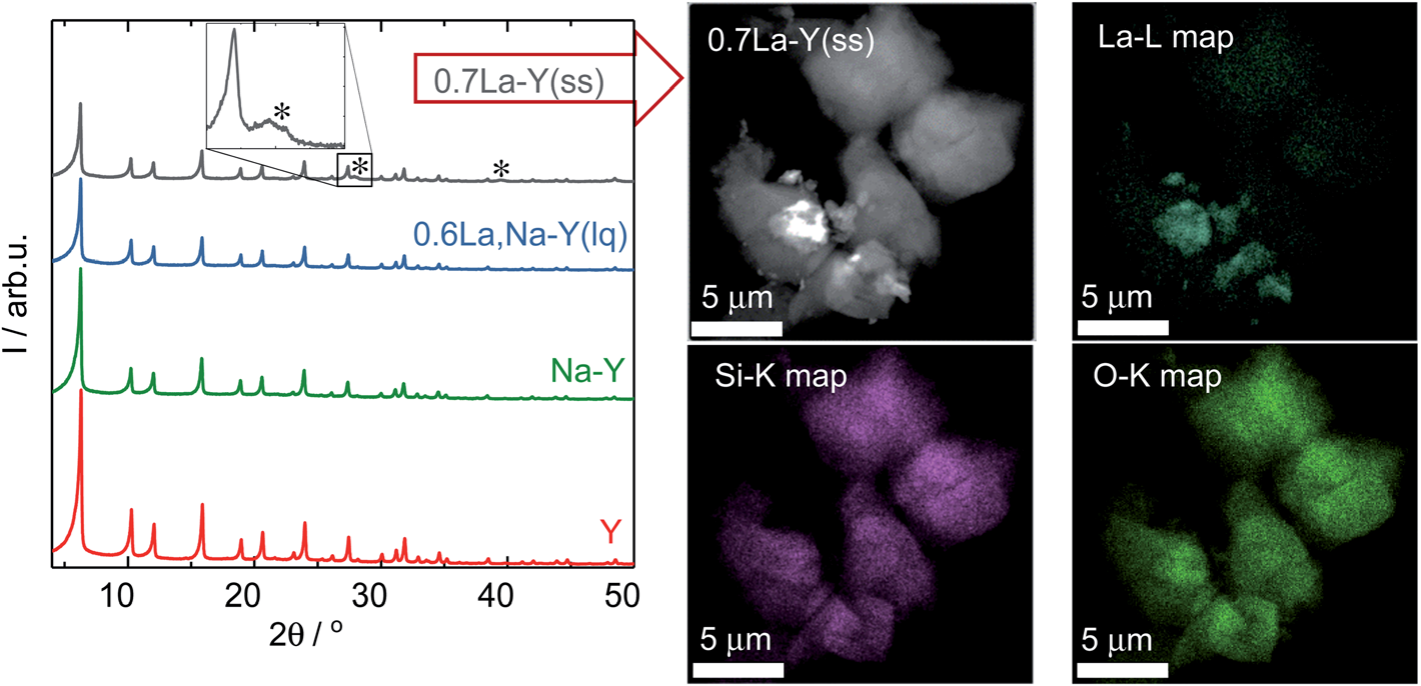
XRD patterns (left) of the zeolites Y, Na–Y, 0.6La,Na–Y(lq) and 0.7La–Y(ss) with asterisks marking diffraction reflections of La_2_O_3_. HAADF-STEM image and corresponding element maps (La–L, Si–K and O–K) of 0.7La–Y(ss) (right).

Regarding the influence of the La^3+^ cation exchange on the structure of Y-type zeolites, the obtained materials were investigated by XRD and DRIFTS. The XRD patterns of Y, Na–Y, 0.6La,Na–Y(lq) and 0.7La–Y(ss) are exemplarily displayed in Fig. 2. Zeolite Y and Na–Y exhibit identical XRD patterns suggesting that the crystal structure of Y-type zeolite stays intact after the Na^+^ cation exchange. Similarly, regardless of the ion exchange procedure applied, the crystallinity of zeolite Y is largely preserved for all obtained materials, even for the zeolites with high La^3+^ cation exchange degrees, *i.e.*, 0.6La,Na–Y(lq) and 0.7La–Y(ss). In agreement with XRD results, DRIFT spectra in the framework vibration region (1300–600 cm^−1^) of 0.7La,Na–Y(lq) and 0.7La–Y(ss) show no significant change in comparison to the parent zeolite Y (*cf.* Fig. S4†). The overall shape and wavenumbers of the bands corresponding to the asymmetric (*ν*_as_ = 1203, 1076 cm^−1^) and symmetric (*ν*_s_ = 830, 792 cm^−1^) stretching vibrations of TO_4_ (T = Si or Al) remain unchanged.

Additionally, the La^3+^ cations were observed in the form of La_2_O_3_ as suggested by the XRD patterns of the zeolite 0.7La–Y(ss) shown in Fig. 2. In particular, two broad reflexes are found at 2*θ* of 27.9° and 39.4° which can be assigned to (100) and (102) planes characteristic of La_2_O_3_ (JCPDS card number 05-0602). However, La_2_O_3_ reflexes are not present for other materials having comparable values of *n*_La_/*n*_Al_ (0.6La,Na–Y(lq) and 0.7La,Na–Y(lq)) from the La^3+^ cation exchange in the liquid phase. Thus, it is suggested that La_2_O_3_ species introduced by the ion exchange procedure in the liquid phase are either present as fine particles (<5 nm) which are not detected by XRD or absent. The observation of La_2_O_3_ particles only after La^3+^ cation exchange in the solid-state for the zeolite 0.7La–Y(ss) with the particle size up to 2 μm (estimated from STEM in Fig. 2, right) might explain its considerably higher standard deviation of La content (Δ*n*_La_ = 0.29 mmol g^−1^) (*cf.* Chapter 3.1.1).

Therefore, it is concluded that both La^3+^ cation exchange procedures hardly affect the zeolite Y crystal structure. However, at high La content (*n*_La_ > 0.50 mmol g^−1^), the comparatively low homogeneity of local La distribution (indicated by the La_2_O_3_ XRD reflexes and a higher Δ*n*_La_ determined by SEM-EDX) might be a drawback of the solid-state La^3+^ cation exchange.

#### 3.1.3 Textural properties

**Fig. 3:**
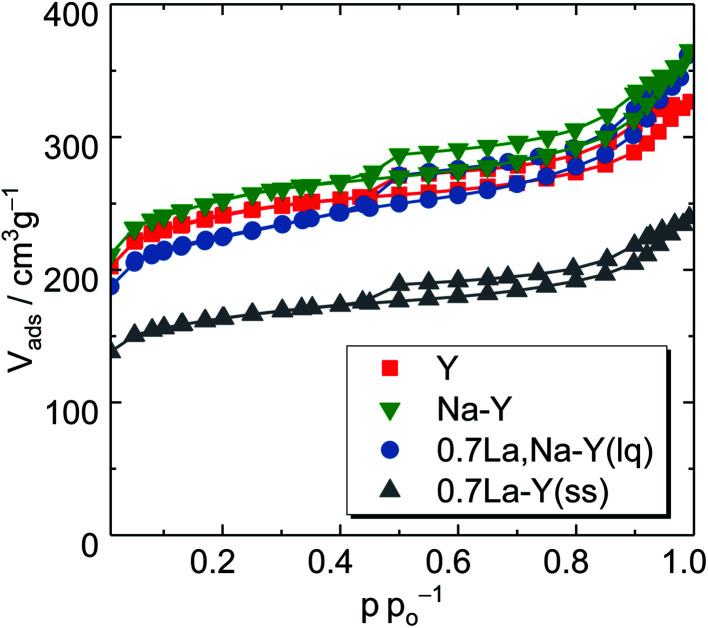
N_2_ sorption isotherms of the zeolites Y, Na–Y and highly La^3+^ cation-exchanged zeolites (0.7La,Na–Y(lq) and 0.7La–Y(ss)).

Furthermore, the textural changes of the zeolites Y, Na–Y after La^3+^ cation exchange were investigated by N_2_ sorption analysis. The obtained N_2_ sorption isotherms of the zeolites Y, Na–Y, 0.7La,Na–Y(lq) and 0.7La–Y(ss) are exemplarily shown in Fig. 3. Both zeolites Y and Na–Y exhibit essentially the same N_2_ sorption isotherm confirming that the Na^+^ cation exchange leaves the pore structure of zeolite Y unaffected. Regardless of the La^3+^ cation exchange applied, a combined type I and type IV isotherm was observed for all obtained materials suggesting that the initial bimodal pore structure of the commercial zeolite Y remained. On the other hand, at the same *n*_La_/*n*_Al_-ratio (0.7), the specific micropore volume after the La^3+^ cation exchange in the liquid phase is slightly reduced for 0.7La,Na–Y(lq) as the N_2_ uptake at *p p*^−1^_0_ < 0.01 is shifted by only 15 cm^3^ g^−1^. However, the specific micropore volume of the 0.7La–Y(ss) is much lower as indicated by the shift of 50 cm^3^ g^−1^ in the N_2_ adsorbed volume at p *p*^−1^_0_ < 0.01 as compared with the parent zeolite Y.

**Fig. 4:**
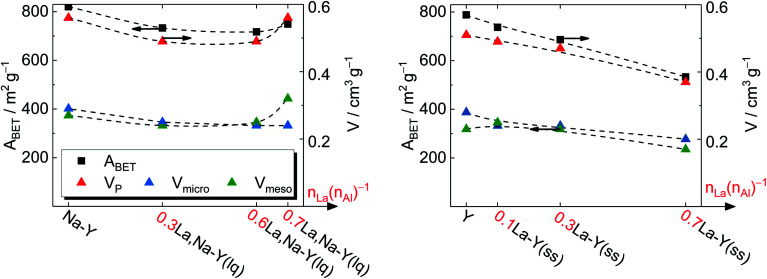
Specific surface area *A*_BET_ and specific pore volume *V*_P_ of zeolite Y before and after La^3+^ cation exchange in the liquid phase (left) and in the solid state (right).

To gain a deeper insight, the *t*-plot model was used to determine the specific micropore volume (*V*_micro_). All absolute values of textural properties, *e.g.*, specific surface area (*A*_BET_), total pore volume (*V*_P_) and *V*_micro_, derived from N_2_ sorption results are summarized in Table 2. Irrespective of the ion exchange procedure applied, *V*_micro_ gradually decreases with increasing *n*_La_/*n*_Al_ up to 0.7 (Fig. 4). As for the solid-state La^3+^ cation exchange, the reduction of *V*_micro_ (from 0.28 to 0.20 cm^3^ g^−1^) is accompanied with a steady loss in specific pore volume *V*_P_ (from 0.51 to 0.37 cm^3^ g^−1^) as well as in specific surface area (from 788 to 533 m^2^ g^−1^). This is probably due to a partial pore blockage caused by La_2_O_3_ particles (*cf.* Chapter 3.1.1).

**Table 2:** Specific surface area (*A*_BET_), specific pore volume (*V*_P_), specific micropore volume (*V*_micro_) and La^3+^ cation exchange degree of the zeolites Y, Na–Y and the La^3+^ cation-exchanged zeolites before (in brackets) and after the test for hydrothermal stability (*c*_LA_ = 0.2 mol L^−1^, *c*_FA_ = 0.6 mol L^−1^, autogenous pressure, 473 K, 24 h)

Material	La^3+^ cation exchange degree/%	*A*_BET_ a/m^2^ g^−1^	*V*_P_ b/cm^3^ g^−1^	*V*_micro_ c/cm^3^ g^−1^
Y	n. d.^d^	113 (788)	0.35 (0.51)	0.00 (0.28)
Na–Y	n. d.	n. d. (815)	n. d. (0.56)	n. d. (0.27)
0.3LaNa–Y(lq)	(62)	124 (733)	0.46 (0.49)	0.01 (0.25)
0.6LaNa–Y(lq)	(130)	212 (717)	0.51 (0.49)	0.04 (0.24)
0.7LaNa–Y(lq)	(104)	145 (749)	0.40 (0.56)	0.02 (0.24)
0.1La–Y(ss)	(21)	n. d. (686)	n. d. (0.47)	n. d. (0.24)
0.3La–Y(ss)	(65)	106 (727)	0.42 (0.49)	0.01 (0.25)
0.7La–Y(ss)	(140)	187 (533)	0.38 (0.37)	0.05 (0.20)
Silylated Y^e^	n. d.	346 (411)	0.31 (0.34)	0.12 (0.15)

a *Via* BET.

b Single point BET.

c *t*-Plot.

d n. d.: not determined.

e Data from ref. 41.

On the other hand, the reduction of *V*_micro_ caused by the La^3+^ cation exchange in the liquid phase is a result of demetallation (*cf.* Chapter 3.1.1). The removal of both Al and Si gradually increases with repeating the ion exchange steps. Consequently, it causes a partial extraction of material from the zeolite and results in a widening of the channels and thus a distinct formation of mesopores. After the third ion exchange step, the effect of demetallation is especially pronounced for the zeolite 0.7La,Na–Y(lq) as indicated by a lower *V*_micro_ (0.24 cm^3^ g^−1^) and a significantly higher specific mesopore volume (*V*_meso_ = 0.32 cm^3^ g^−1^) with respect to the parent zeolite Y (*V*_micro_ = 0.28 cm^3^ g^−1^, *V*_meso_ = 0.23 cm^3^ g^−1^).

Comparing the extent of influence of the two La^3+^ cation exchange steps (in the liquid and the solid phase) on the textural properties, the procedure in solid state leads to a higher loss in *V*_micro_, *i.e.*, 0.08 cm^3^ g^−1^ (0.7La–Y(ss)) *vs.* 0.04 cm^3^ g^−1^ (0.7La,Na–Y(lq)), and also in *A*_BET_ (39 m^2^ g^−1^*vs.* 255 m^2^ g^−1^, respectively). However, the solid-state La^3+^ cation exchange still is more attractive due to the reasons stated in Chapter 3.1.1 (mainly the fact that less La salt is needed to achieve comparable La contents).

### 3.2 Hydrothermal stability investigation

**Fig. 5:**
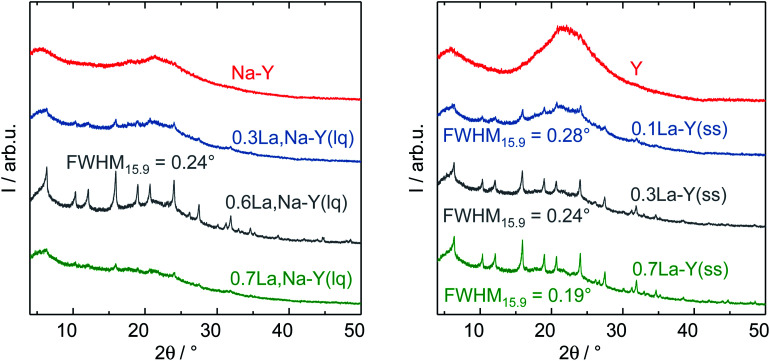
XRD patterns with corresponding FWHM_15.9_ in boxes for the zeolites Y, Na–Y and the La^3+^ cation-exchanged zeolite Y in the liquid phase (left) and in the solid state (right) after the hydrothermal stability test.

The zeolites Y, Na–Y and the La^3+^ cation-exchanged zeolites were subjected to a test to examine the influence of introduction of La^3+^ cations and the procedure of La^3+^ cation exchange on their hydrothermal stability under APP conditions (*cf.* Chapter 2.4). Table 2 shows results of N_2_ sorption analysis of the zeolites after the stability test for 24 h. In particular, zeolite Y underwent a substantial structural disintegration as can be seen from the complete loss of micropore volume and a significant decrease in *A*_BET_ (86%). This is in good agreement with the loss of characteristic reflexes of Y-type zeolite and the observation of a broad signal at 2*θ* of 21° representative for an amorphous silica phase in the corresponding XRD pattern (Fig. 5). A similar XRD pattern was also recorded for Na–Y after the stability test suggesting that the Na^+^ cation exchange had a negligible influence on the hydrothermal stability of zeolite Y.

Independent of the ion exchange procedure, La^3+^ cation-exchanged zeolites show a partial preservation of the initial textural properties and the faujasite-type structure after the hydrothermal stability test as indicated by XRD results (Fig. 5). The N_2_ sorption results suggest that the preservation extent of *A*_BET_, *V*_P_ and *V*_micro_ is associated with the La^3+^ cation exchange degree. With respect to the La^3+^ cation exchange in the liquid phase, the increase in the La^3+^ cation exchange degree from 62% to 104%, 130% results in a smaller loss in *A*_BET_ (83%, 81% and 70%) and *V*_micro_ (96%, 92% and 83%). Thus, 0.6La,Na–Y(lq) with the highest La^3+^ cation exchange degree of 130% is the most stable among the La^3+^ cation-exchanged zeolites based on Na–Y. This finding is consistent with those from XRD (Fig. 5, left). The reflexes characteristic of faujasite-type zeolites are still clearly visible in the XRD pattern of 0.6La,Na–Y(lq), however, less pronounced for 0.7La,Na–Y(lq) and 0.3La,Na–Y(lq).

Similar to the observation for the La^3+^ cation exchange in the liquid phase, 0.7La–Y(ss) (La^3+^ cation exchange degree = 140%) is the most stable material obtained from the solid-state La^3+^ cation exchange procedure. In comparison to 0.6La,Na–Y(lq), 0.7La–Y(ss) exhibits the smaller loss of *A*_BET_ (65% *vs.* 70%) and *V*_micro_ (75% *vs.* 83%). Thus, 0.7La–Y(ss) is the most stable zeolite among all materials investigated.

Furthermore, the full width at half maximum (FWHM) of XRD reflexes, which is inversely proportional to the crystallite size of zeolite Y, was used as a measure to (semi-) quantitatively determine the extent of structural degradation after test of hydrothermal stability. To mitigate the axial divergence yielding asymmetric reflexes and reducing the calculation inaccuracy, which are more pronounced at low Bragg angles, the second prominent reflex centering at 2*θ* = 15.9° was investigated. The full width at half maximum of the reflex at 2*θ* = 15.9° is referred to as FWHM_15.9_. Zeolite Y exhibits a FWHM_15.9_ of 0.09° which subsequently increases to 0.19° (0.7La–Y(ss)-140%), 0.24° (0.3La–Y(ss)-65% or 0.6La,Na–Y(lq)-130%) and 0.28° (0.1La–Y(ss)-21%) after the stability test (Fig. 5, right). The increase in FWHM is indicative of a decline in crystallite size probably due to framework disintegration. Therefore, it is confirmed that 0.7La–Y(ss) with the smallest FWHM_15.9_ is the most stable among all investigated materials.

Noticeably, 0.3La–Y(ss) and 0.6La,Na–Y(lq) exhibit an identical FWHM_15.9_ (0.24°) despite of the difference in La^3+^ cation exchange degree (65% *vs.* 130%, respectively). The similar stability despite different ion exchange degrees might be related to a demetallation taking place during the preparation of 0.6La,Na–Y(lq) which did not occur for 0.3La–Y(ss) (*cf.* Chapter 2.2.1). The removal of Si and Al generates additional framework defects, *i.e.*, silanol groups, which may facilitate the structural degradation.

In comparison to a zeolite Y silylated using trichloromethylsilane (silane-to-zeolite ratio of 10 mol g^−1^) reported earlier^41^ (later referred to as silylated Y), 0.7La–Y(ss) exhibits a lower resistance towards structural degradation. The higher susceptibility to the water attack of 0.7La–Y(ss) is evident from its XRD pattern with a higher FWHM_15.9_ (0.19° (Fig. 5)), compared with 0.13° of silylated zeolite Y (*cf.* Fig. S6†) suggesting less pronounced structural disintegration for silylated zeolite Y after 24 h of treatment. In line with XRD results, N_2_ sorption analysis of silylated zeolite Y shows a smaller loss in *V*_micro_ (20% *vs.* 75%) and in *A*_BET_ (16% *vs.* 65%, Table 2) in comparison to 0.7La–Y(ss) in the current study.

**Fig. 6:**
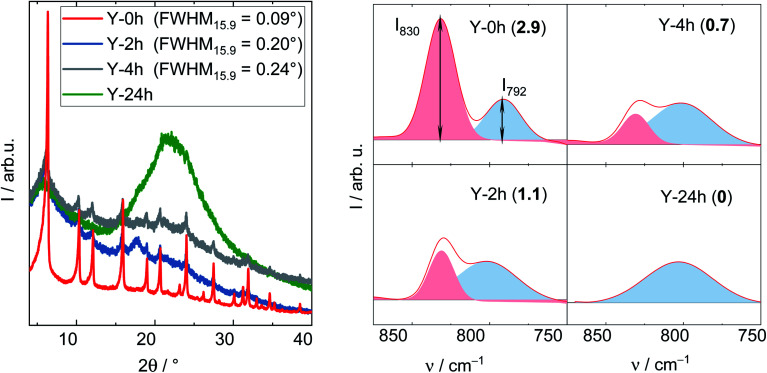
XRD patterns (left) and corresponding deconvoluted DRIFT spectra recorded at 373 K (right) with the number in bracket showing *I*_830_/*I*_792_ of zeolite Y after the exposure to the stability test for 2,4 and 24 h (*c*_LA_ = 0.2 mol L^−1^, *c*_FA_ = 0.6 mol L^−1^, 493 K, *n* = 700 min^−1^). For clarity, the XRD pattern of zeolite Y was offset.

The degradation of zeolite Y framework under APP conditions was further examined by DRIFTS. Firstly, a series of parent zeolite Y with different degradation degree was prepared by varying the treatment time (2, 4 and 24 h) under reaction conditions typical for APP (*c*_LA_ = 0.2 mol L^−1^, *c*_FA_ = 0.6 mol L^−1^, autogenous pressure, 493 K, 24 h, *n* = 700 min^−1^). The obtained materials were characterized by XRD, DRIFTS (Fig. 6) and N_2_ adsorption analysis (*cf.* Table S3†). In the XRD patterns, the FWHM_15.9_, as expected, increases from 0.09° (Y) to 0.20° (after 2 h), 0.24° (after 4 h) and is eventually indistinct from the background with a broad signal at 2*θ* of 21° representative for amorphous silica (after 24 h). Accordingly, a gradual decrease in *A*_BET_ was also observed with prolonging the treatment time, in particular from 788 m^2^ g^−1^ (Y) to 461, 260 and 111 m^2^ g^−1^ after 2, 4 and 24 h, respectively. These observations suggest that the drastic crystallinity loss and amorphization of the zeolite Y occurs quickly during the first 2 h of the experiment. The structural disintegration was complete after 24 h. The same phenomenon is also observed using DRIFT spectroscopy. Here, the shape of symmetric stretching vibration band (*ν*_s_ = 850–750 cm^−1^) changes accordingly with increasing the treatment time. To properly interpret the *ν*_s_ band, deconvolution was applied resulting in a group of two compositional bands centered at approximately 830 and 792 cm^−1^. The former band is assigned to the “inter-tetrahedral” mode, which is structure-sensitive, *i.e.*, susceptible to framework distortion affecting the long-range order, whereas the latter at 792 cm^−1^ corresponds to the “intra-tetrahedral” mode and is structure-insensitive.^49^ Indeed, the intensity of the band centered at 830 cm^−1^ (later on referred to as *I*_830_) gradually weakens with increasing the treatment time. Furthermore, the band at 830 cm^−1^ is no longer observed after 24 h, at which the amorphization was complete. In contrast, the intensity of the band at 792 cm^−1^, *i.e.*, *I*_792_, remained unchanged even after 24 h reaction time. Therefore, the *I*_830_/*I*_792_ ratio was used as a measure of the degradation degree of zeolite Y framework. The results are consistent with XRD, N_2_ adsorption and DRIFT analysis. With increasing the hydrothermal treatment time, *i.e.*, a higher degree of structural disintegration, *I*_830_/*I*_792_ ratio decreases significantly from 2.9 (Y-0 h) to 1.1 (Y-2 h), 0.7 (Y-4 h) and further to 0 (Y-24 h). Therefore, it is concluded that DRIFTS, employed in this function for the first time in this study, can be used as a supplementary measure to quantify the degradation degree of zeolite Y framework after hydrothermal treatment.

**Fig. 7:**
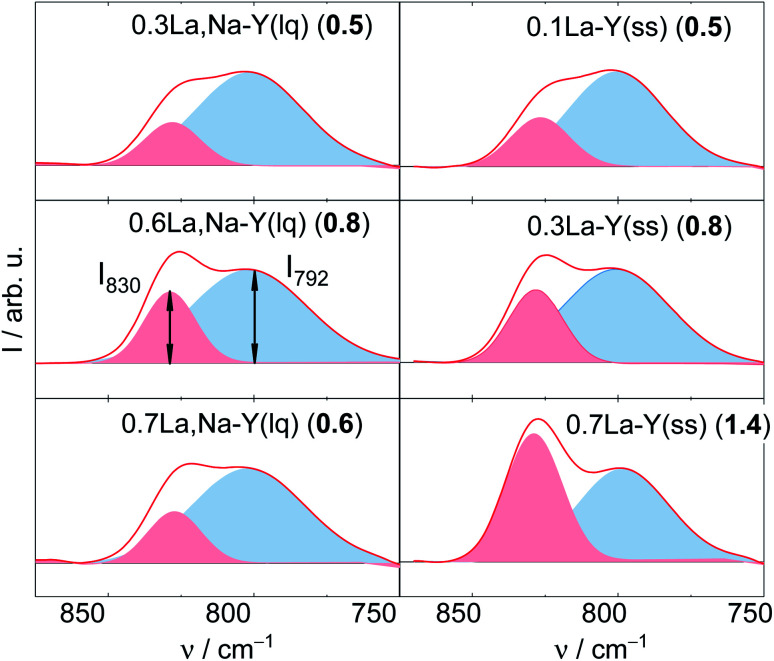
Deconvoluted DRIFT spectra (recorded at 373 K) with corresponding *I*_830_/*I*_792_ (in brackets) of La^3+^ cation-exchanged Y after the stability investigation (*c*_LA_ = 0.2 mol L^−1^, *c*_FA_ = 0.6 mol L^−1^, autogenous pressure, 473 K, 24 h).

The deconvoluted DRIFTS data of all investigated samples after the stability test are shown in Fig. 7. Accordingly, all La^3+^ cation-exchanged zeolites can be placed in a descending order of the hydrothermal stability with the corresponding *I*_830_/*I*_792_ (after 24 h of treatment time): 0.7La–Y(ss) (1.4) > 0.6La,Na–Y(lq) (0.8) ∼0.3La–Y(ss) (0.8) > 0.7La,Na–Y(lq) (0.6) > 0.1La–Y(ss) ∼0.3La,Na–Y(lq) (0.5) > Y (0.0). This finding confirms that 0.7La–Y(ss) is the most stable among the investigated materials. It was thus selected for further investigation of the catalytic activity for the hydrogenation of LA in the presence of FA.

### 3.3 Hydrogenation of levulinic acid

After loading with Pt, 2.7Pt/Y and 2.6Pt/0.7La–Y(ss) the most stable zeolites from Chapter 3.2, were applied as bifunctional catalysts in the hydrogenation of LA with formic acid (FA) as internal hydrogen source. Note that the introduction of Pt leaves the textural properties of the zeolite essentially unaffected as evident, for instance, for the catalyst 2.7Pt/Y with a specific surface area of *A*_BET_ = 775 m^2^ g^−1^ before (Y, *cf.*Table 2) and 788 m^2^ g^−1^ (2.7Pt/Y) after loading with Pt. This is also in accordance with the presence of small Pt nanoparticles on the catalyst.

**Fig. 8:**
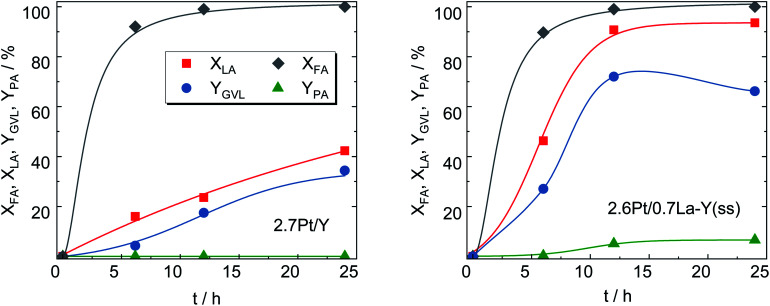
LA and FA conversion (*X*_LA_, *X*_FA_), GVL and PA yield (*Y*_GVL_, *Y*_PA_) over 2.7Pt/Y and 2.6Pt/0.7La–Y(ss) (reaction conditions: *m*_cat_ = 0.5 g, *V* = 125 cm^3^, *c*_LA_ = 0.2 mol L^−1^, *c*_FA_ = 0.6 mol L^−1^, autogenous pressure, 493 K, 24 h, *n* = 700 min^−1^).

Nearly identical conversion over time was observed for both catalysts in the decomposition of FA (for H_2_-generation) as depicted in Fig. 8. This is an indication for similar properties of the deposited Pt species catalyzing this reaction and is further confirmed by the comparable Pt content of both catalysts determined by ICP-OES (results shown in Table 3).

Interestingly, despite significantly lower specific surface area (533 *vs.* 788 m^2^ g^−1^), 2.6Pt/0.7La–Y(ss) displays a notably higher LA hydrogenation activity, *i.e.*, *X*_LA_ = 94%, in comparison to 42% for 2.7Pt/Y after a reaction time of 24 h. Sampling during 24 h of reaction time revealed that LA conversion and GVL yield linearly increased up to 42% and 34%, respectively, for 2.7Pt/Y. In contrast, 2.6Pt/0.7La–Y(ss) displayed a higher LA conversion (94%) and a significantly increased GVL yield (72%) after 12 h. This increase in catalytic activity of 2.6Pt/0.7La–Y(ss) over 2.7Pt/Y, assuming similar properties of the Pt-phase (see above), can be attributed to either a change in acid properties or the improved hydrothermal stability of 2.6Pt/0.7La–Y(ss). Both catalysts investigated in the hydrogenation of LA (2.7Pt/Y and 2.6Pt/0.7La–Y(ss)) show similar acid properties as exemplified by an analogous acid site density (ASD), *i.e.*, 360 μmol g^−1^ (2.7Pt/Y) and 369 μmol g^−1^ (2.6Pt/0.7La–Y(ss)) (Table 3). In addition, the BAS-to-LAS ratio determined by DRIFTS is 2.0 (2.7Pt/Y) and 1.4 (2.6Pt/0.7La–Y(ss)) suggesting a comparable fraction of BAS which are required for the LA conversion (*cf.* Fig. S8†). In agreement, TPD-NH_3_ results show only a slight decrease in the strong acid site fraction (*T*_des_ ≥ 750 K) of 2.6Pt/0.7La–Y(ss), *i.e.*, 11%, compared with 14% of 2.7Pt/Y. The reduction of strong acid sites for 2.6Pt/0.7La–Y(ss) is probably a result of dehydroxylation and the increasing fraction of weak acid sites (*T*_des_ ≤ 500 K) corresponding to incorporated La^3+^ cations, *i.e.*, 35% for 2.6Pt/0.7La–Y(ss) compared with 10% for 2.7Pt/Y. Thus, in the current study it is assumed that the acid properties of 2.7Pt/Y and 2.6Pt/0.7La–Y(ss) have inconsiderable influence on the hydrogenation of LA to GVL. Since a loss of 41% *A*_BET_ and 51% *V*_micro_ was observed for zeolite Y quickly after the first 2 h of reaction time (*cf.* Y-2 h, Table S3†), the reduced catalytic activity of 2.7Pt/Y is attributed to its lower hydrothermal stability and the resulting substantial structural disintegration. To further validate this hypothesis, the zeolite 0.7La–Y(ss) was applied in the hydrogenation of LA under identical reaction conditions for 2 h (*c*_LA_ = 0.2 mol L^−1^, *c*_FA_ = 0.6 mol L^−1^, autogenous pressure, 493 K, *n* = 700 min^−1^). After filtration, the obtained solid was characterized by DRIFTS and XRD (*cf.* Fig. S9†). In comparison to Y, 0.7La–Y(ss) exhibits a higher ratio of *I*_830_/*I*_792_ (1.4 *vs.* 1.1) and a smaller FWHM_15.9_ (0.17° *vs.* 0.20°) confirming higher preservation of the framework for 0.7La–Y(ss). This is in agreement with the N_2_ sorption results of the catalysts 2.7Pt/Y and 2.6Pt/0.7La–Y(ss) after the hydrogenation of LA with FA for 24 h (Fig. S11†). Both catalysts exhibit mostly mesopores which correspond to a specific mesopore volume of 0.35 cm^3^ g^−1^ (2.7Pt/Y) and 0.20 cm^3^ g^−1^ (2.6Pt/0.7La–Y(ss), respectively. The lower extent of mesopore formation after the catalytic investigation of 2.6Pt/0.7La–Y(ss) again confirms its higher hydrothermal stability. Hence, the enhanced hydrothermal stability by solid-state La^3+^ cation exchange is assumed to be the reason for the improved catalytic activity of 2.6Pt/0.7La–Y(ss) (*X*_LA_, = 93%, *Y*_GVL_ = 66%) as compared to 2.7Pt/Y (*X*_LA_, = 42%, *Y*_GVL_ = 34%) after 24 h reaction time.

**Table 3:** Pt content (*ω*_Pt_/wt%), acid site density (ASD) and specific surface area (*A*_BET_) of 2.7Pt/Y and 2.6Pt/0.7La–Y(ss) applied in the hydrogenation of LA with FA (reaction conditions: *m*_cat_ = 0.5 g, *V* = 125 cm^3^, *c*_LA_ = 0.2 mol L^−1^, *c*_FA_ = 0.6 mol L^−1^, autogenous pressure, 493 K, 24 h, *n* = 700 min^−1^)

Catalyst	*ω*Pt^a^/wt%	ASD^b^/μmol g^−1^	*A*_BET_/m^2^ g^−1^	*X*_FA_/%	*X*_LA_/%	*Y*_GVL_/%	*Y*_PA_/%
2.7Pt/Y	2.7	360	788	100	42	34	0
2.6Pt/0.7La–Y(ss)	2.6	369	533	100	93	66	7
2.5Pt/silylated Y^d^	2.4	n. d.^c^	411	100	38	12	0

a From ICP-OES.

b From TPD-NH_3_.

c n. d.: not determined

d Data from ref. 41.

**Scheme 2:**
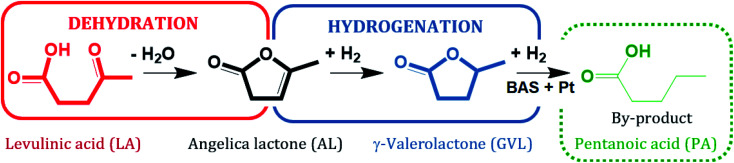
Reaction pathway of the hydrogenation of LA with FA over 2.6Pt/0.7La–Y(ss) at 493 K.

Not only the catalytic activity for the hydrogenation of LA into GVL was improved by using 2.6Pt/0.7La–Y(ss). This catalyst also facilitates the formation of pentanoic acid (PA), *i.e.*, *Y*_PA_ = 7%, which was not observed as a reaction product using 2.7Pt/Y. PA is a product of consecutive hydrogenation *via* the thermodynamically disfavored ring-opening of GVL which strongly depends on the presence of active metal and acid sites^50–52^ (Scheme 2). The observation of PA as a reaction product of the hydrogenation of LA using 2.6Pt/0.7La–Y(ss) suggests that the incorporation of La^3+^ cations increases the hydrothermal stability and by that preserves the activity of the metal/acid sites of 2.6Pt/0.7La–Y(ss) under reaction conditions for a longer time, thus enabling the consecutive reaction of GVL to PA to happen.

Finally, the results obtained in the hydrogenation of LA with the La^3+^ cation-exchanged zeolite Y were compared with the results from an earlier study where the hydrothermal stability was improved *via* silylation. Despite being more susceptible to the structural degradation (*cf.* Chapter 3.2, Table 2), 2.6Pt/0.7La–Y(ss) showed a higher catalytic activity, *e.g.*, *Y*_GVL_ = 66%, as compared with *Y*_GVL_ = 12% for 2.5Pt/silylated Y catalyst under identical reaction conditions (*c*_LA_ = 0.2 mol L^−1^, *c*_FA_ = 0.6 mol L^−1^, 493 K, *p* ∼ 4 MPa and 24 h). The low catalytic activity of 2.5Pt/silylated Y is mainly attributed to the limited accessibilities to active sites due to the undesired pore blockage by the polymeric silylation layer (as also evident from the lower observed *A*_BET_ (411 m^2^ g^−1^ for 2.5/silylated Y as compared to 788 m^2^ g^−1^ for 2.7Pt/Y). In addition, the silylation increases the hydrophobicity of the catalyst further reducing the concentration of the reactants in the proximity to the active sites. Therefore, solid-state La^3+^ cation exchange can be considered more suitable than silylation for improving hydrothermal stability and the resulting catalytic activity of bifunctional zeolite Y catalysts for the aqueous-phase hydrogenation of LA with FA.

## 4. Conclusions

La^3+^ cation exchange of zeolite Y in either liquid phase or solid state is demonstrated to mitigate the framework disintegration under hydrothermal conditions as applied in aqueous phase processing of biomass (water as solvent, pH ∼2, 473–493 K, *p* ∼4 MPa). The stabilization effect is proportional with the La^3+^ cation exchange degree irrespective of the ion exchange procedure applied. Consequently, the material with the highest La^3+^ cations exchange degree, 0.7La–Y(ss) (140%), was the most stable among all studied materials. In order to obtain highly La^3+^ cation-exchanged faujasite-type zeolites (*n*_La_ ≥ 0.5 mmol g^−1^), the solid-state La^3+^ cation exchange is advantageous as no sequential ion exchange experiments are needed and the consumption of expensive La salt is greatly reduced. More importantly, the demetallation generating additional framework defects is avoided as compared to the procedure in liquid phase. However, a lower homogeneity of the local La distribution might be a drawback of solid-state La^3+^ cation exchange. After impregnation with Pt the bifunctional 2.6Pt/0.7La–Y(ss) catalyst is significantly more active as catalyst in the hydrogenation of LA after 24 h, *i.e.*, *X*_LA_ = 94%, *Y*_GVL_ = 66%, compared to the unmodified 2.7Pt/Y catalyst (*X*_LA_ = 42%, *Y*_GVL_ = 34%). This increase in activity is likely attributed to the improved hydrothermal stability of the Y-type zeolite. Despite being more susceptible to framework disintegration than the silylated catalyst reported earlier, the La^3+^ cation exchanged catalyst shows a higher activity in the hydrogenation of LA than its silylated counterpart (*X*_LA_ = 38% and *Y*_GVL_ = 12%). This is attributed to limited accessibility to the active sites due to silylation. Furthermore, DRIFTS using the intensity ratio of the bands at 830 and 792 cm^−1^ is, for the first time, proven to be a useful supplementary tool, in addition to the widely accepted methods, *e.g.*, XRD and N_2_ sorption, to quantify the structural disintegration of zeolite Y by hydrothermal treatment.

## Conflicts of interest

There are no conflicts of interest to declare.

## Supplementary Material

RA-011-D0RA08907A-s001
