# Supplementary material for: Improving the hydrothermal stability of zeolite Y by La3+ cation exchange as a catalyst for the aqueous-phase hydrogenation of levulinic acid
Source: RSC Adv. 2021 Jan 29;11(10):5568–79. doi: 10.1039/d0ra08907a (PMC8694756; doi:10.1039/d0ra08907a)
Supplement: RA-011-D0RA08907A-s001 [file RA-011-D0RA08907A-s001.pdf]

Electronic Supplementary Material (ESI) for RSC Advances.
This journal is © The Royal Society of Chemistry 2021

Supplementary Information

Improving the Hydrothermal Stability of Zeolite Y by La^{3+} Cation Exchange as a Catalyst for the Aqueous-Phase Hydrogenation of Levulinic Acid

*Hue-Tong Vu, Michael Goepel, Roger Gläser**

Institute of Chemical Technology, Universität Leipzig, Linnéstr. 3, 04103 Leipzig, Germany

*Corresponding author (roger.glaeser@uni-leipzig.de)

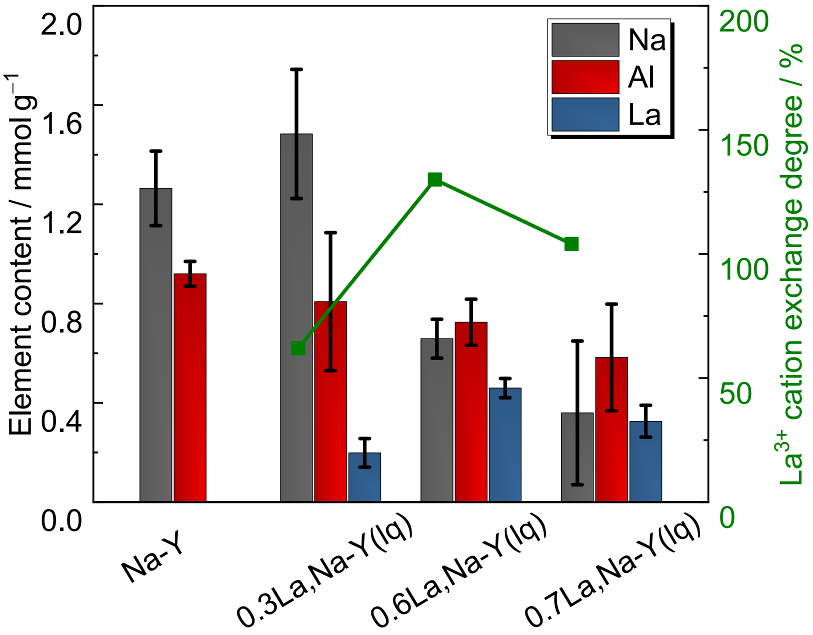

Fig. S.1: Elemental composition and La^{3+} cation exchange degree of zeolite Na-Y before and after La^{3+} cation exchange in the liquid phase, determined by EDX analysis.

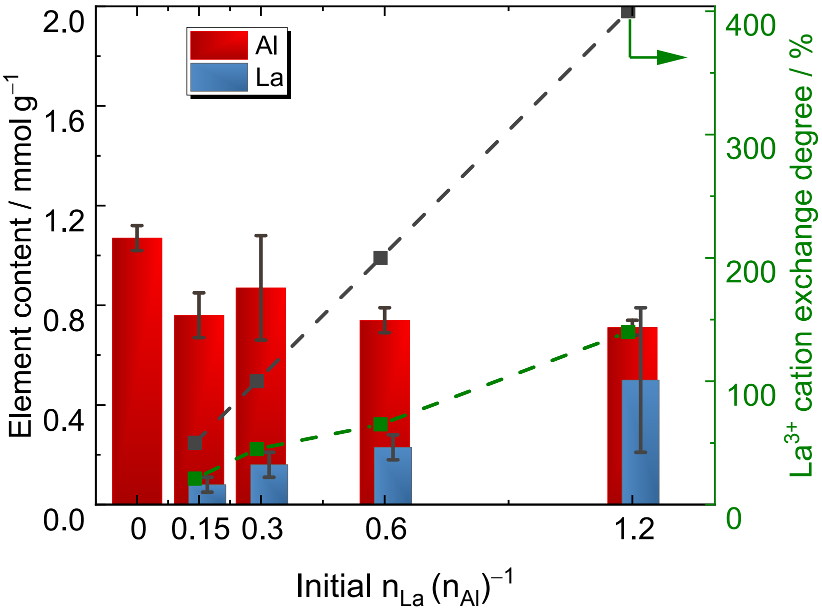

Fig. S.2: Elemental composition and La^{3+} cation exchange degree (at presumably 100% ion exchange efficiency (O) and as obtained (O)) of solid-state La^{3+} cation-exchanged Y, determined by EDX analysis.

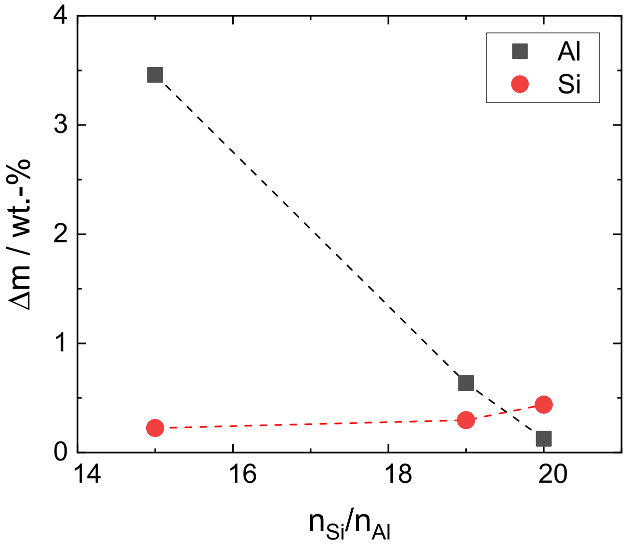

Fig. S.3: Weight percentages of Al (■) and Si (●) leached after three sequential La^{3+} cation exchange experiments in the liquid phase as a function of the n_{Si}/n_{Al}-ratios of the starting materials (Na-Y (n_{Si}/n_{Al} = 15), 0.3La,Na-Y(lq) (n_{Si}/n_{Al} = 19) and 0.6La,Na-Y(lq) (n_{Si}/n_{Al} = 20)).

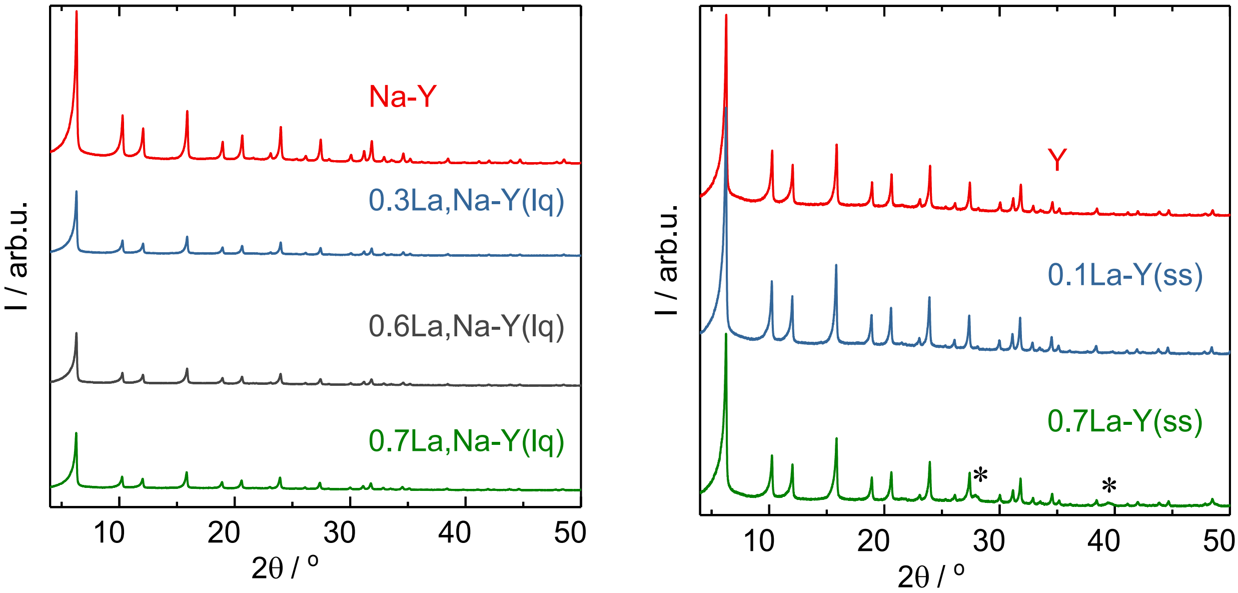

Fig. S.4: XRD patterns of the zeolites Y, Na-Y and La-containing zeolites La^{3+} cation-exchanged in the liquid phase (*x*La,Na-Y(lq), left) or in the solid state (*x*La-Y(ss), right). Asterisks mark La_2O_3 phase.

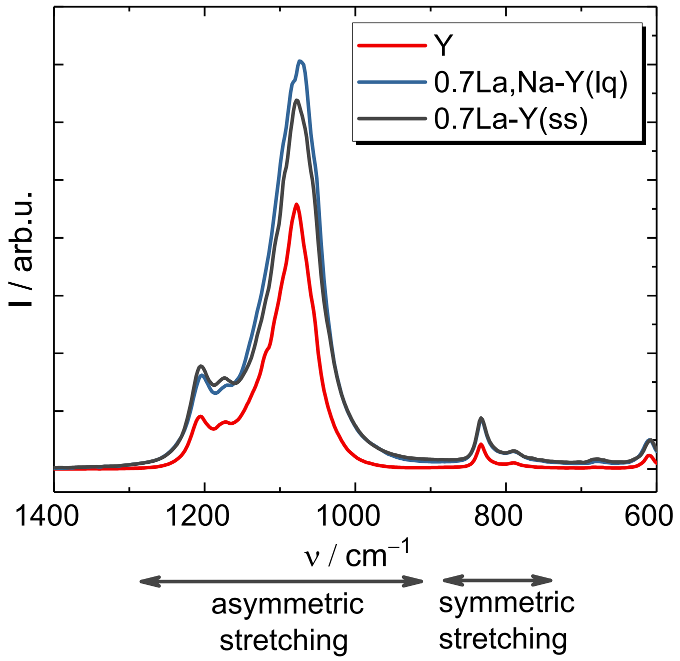

Fig. S.5: DRIFT spectra recorded at 373 K for zeolite Y (red) and the highly La^{3+} cation-exchanged zeolites 0.7La,Na-Y(lq) (blue) and 0.7La-Y(ss) (grey).

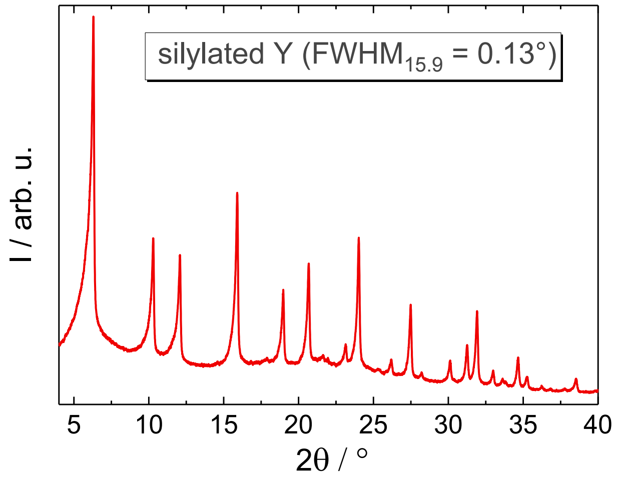

Fig. S.6: XRD pattern of silylated zeolite Y after the stability test (c_{LA} = 0.2 mol L^{-1}, c_{FA} = 0.6 mol L^{-1}, autogenous pressure, 473 K, 24 h), adapted from [1].

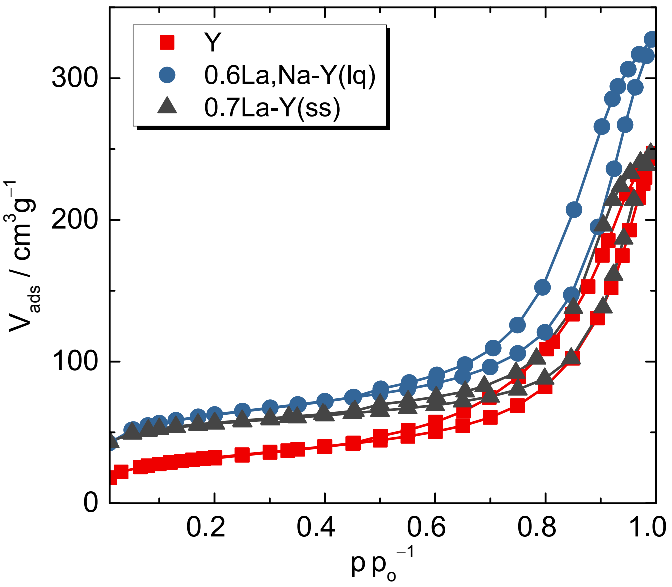

Fig. S.7: N_2 sorption isotherms of zeolite Y (red) and the La^{3+} cation-exchanged zeolites 0.6La,Na-Y(lq) (blue) and 0.7La-Y(ss) (grey) after the stability test (c_{LA} = 0.2 mol L^{-1}, c_{FA} = 0.6 mol L^{-1}, autogenous pressure, 473 K,).

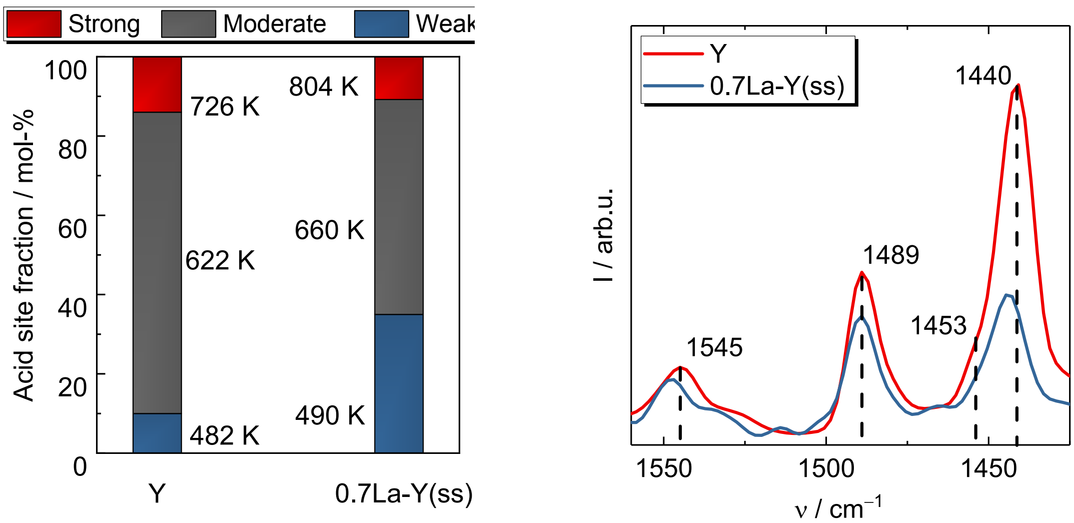

Fig. S.8: Fractions of weak, moderate and strong acid sites obtained from TPD of NH_3 with corresponding temperature at maximum desorption of NH_3 (left) and difference DRIFT spectra of adsorbed pyridine (right) for the zeolites Y and 0.7La-Y(ss). (Strong: $T_{des} \geq 750$ K, weak: $T_{des} \leq 500$ K, moderate: 500 K < T_{des} < 750 K).

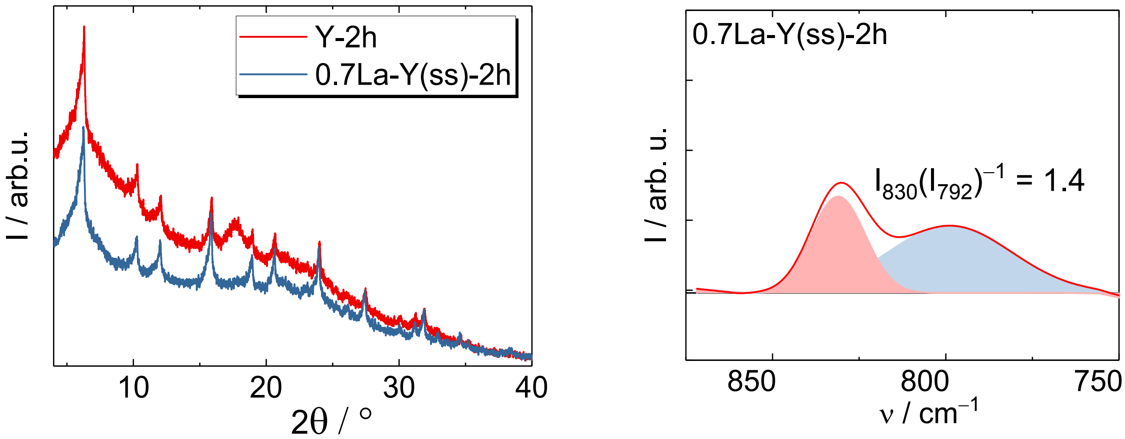

Fig. S.9: XRD patterns of the zeolites Y and 0.7La-Y(ss) after exposure to reaction mixture for hydrogenation of LA with FA for 2 h (reaction conditions: m_{cat} = 0.5 g, V = 125 cm^3, c_{LA} = 0.2 mol L^{-1}, c_{FA} = 0.6 mol L^{-1}, p ≈ 4 MPa, 493 K, n = 700 min^{-1}).

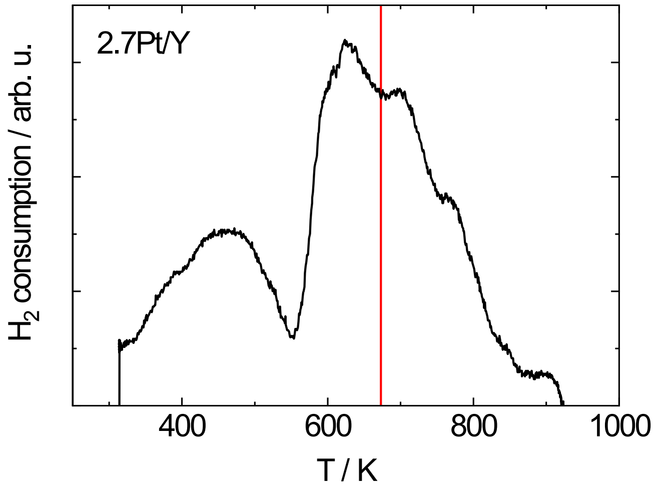

Fig. S.10: TPR profile of 2.7Pt/Y with the red line marking the reduction temperature of 2.7Pt/Y and 2.6Pt/0.7La-Y(ss).

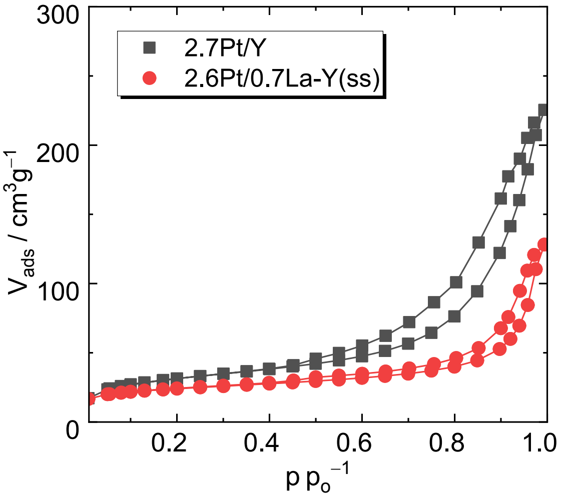

Fig. S.11: N_2 sorption isotherms of 2.7Pt/Y and 2.6Pt/0.7La-Y(ss) after the hydrogenation of levulinic acid with formic acid. Reaction conditions: m_{cat} = 0.5 g, V = 125 cm^3, c_{LA} = 0.2 mol L^{-1}, c_{FA} = 0.6 mol L^{-1}, p ≈ 4 MPa, 493 K, n = 700 min^{-1}.

Tab. S.1: n_{Si}/n_{Al} and n_{La}/n_{Al}-ratios of zeolite Y and La^{3+} cation exchange degree before and

after La^{3+} cation exchange in the liquid phase determined by EDX analysis.

Material	Atomic ratio		La^{3+} cation exchange degree
	n_{Si}/n_{Al}	n_{La}/n_{Al}	/ %
Y	16 ± 1.0	n. d. [a)]	n. d.
0.2La-Y(lq)	20 ± 1.6	0.2 ± 0.1	45
0.3La-Y(lq)	20 ± 2.1	0.3 ± 0.1	55
0.4La-Y(lq)	20 ± 2.7	0.4 ± 0.1	75

a) n. d.: not determined.

Tab. S.2: Starting materials with n_{Si}/n_{Al}-ratios, Al^{3+}, Si^{4+} concentrations of the filtrates and the corresponding weight percentages of Al, Si leached after three sequential La^{3+} cation exchange experiments in the liquid phase.

Starting materials	n_{Si}/n_{Al}	Concentration / mg L^{-1}		Δm / wt.-%	
		Al^{3+}	Si^{4+}	Al	Si
Na-Y	15	3.4	3.6	3.5	0.2
0.3La,Na-Y(lq)	19	0.6	4.7	0.6	0.3
0.6La,Na-Y(lq)	20	0.1	6.9	0.1	0.4

Tab. S.3: Specific surface area (A_{BET}), specific pore volume (V_P) and specific micropore volume (V_{micro}) of zeolite Y before and after exposure to the reaction mixture for hydrogenation of LA with FA at various durations (0, 2, 4 and 24 h, reaction conditions: c_{LA} = 0.2 mol L^{-1}, c_{FA} = 0.6 mol L^{-1}, autogenous pressure (~ 4 MPa), 493 K). The subscripts *micro* indicates micropores.

Material	Time / h	A_{BET} [a)] / $m^2\ g^{-1}$	V_P [b)] / $cm^3\ g^{-1}$	V_{micro} [c)] / $cm^3\ g^{-1}$
Y-0h	0	788	0.51	0.28
Y-2h	2	461	0.62	0.13
Y-4h	4	260	0.46	0.07
Y-24h	24	111	0.35	0.00

a) via BET, b) from single point BET, c) t-plot.
